# A lightweight wheat ear counting model in UAV images based on improved YOLOv8

**DOI:** 10.3389/fpls.2025.1536017

**Published:** 2025-02-11

**Authors:** Ruofan Li, Xiaohua Sun, Kun Yang, Zhenxue He, Xinxin Wang, Chao Wang, Bin Wang, Fushun Wang, Hongquan Liu

**Affiliations:** ^1^ College of Information Science and Technology, Hebei Agricultural University, Baoding, China; ^2^ Department of Digital Media, Hebei Software Institute, Baoding, China; ^3^ Hebei Key Laboratory of Agricultural Big Data, Hebei Agricultural University, Baoding, China; ^4^ College of Horticulture, Hebei Agricultural University, Baoding, China; ^5^ Agricultural Engineering Technology Research Center of National North Mountainous Area, Hebei Agricultural University, Baoding, China; ^6^ College of Urban and Rural Construction, Hebei Agricultural University, Baoding, China; ^7^ State Key Laboratory of North China Crop Improvement and Regulation, Hebei Agricultural University, Baoding, China

**Keywords:** wheat ear detection, unmanned aerial vehicle, small target, YOLOv8, lightweight

## Abstract

Wheat (*Triticum aestivum* L.) is one of the significant food crops in the world, and the number of wheat ears serves as a critical indicator of wheat yield. Accurate quantification of wheat ear counts is crucial for effective scientific management of wheat fields. To address the challenges of missed detections, false detections, and diminished detection accuracy arising from the dense distribution, small size, and high overlap of wheat ears in Unmanned Aerial Vehicle (UAV) imagery, we propose a lightweight model, PSDS-YOLOv8 (P2-SPD-DySample-SCAM-YOLOv8), on the basis of the improved YOLOv8 framework, for the accurate detection of wheat ears in UAV images. First, the high resolution micro-scale detection layer (P2) is introduced to enhance the model’s ability to recognize and localize small targets, while the large-scale detection layer (P5) is eliminated to minimize computational redundancy. Then, the Spatial Pyramid Dilated Convolution (SPD-Conv) module is employed to improve the ability of the network to learn features, thereby enhancing the representation of weak features of small targets and preventing information loss caused by low image resolution or small target sizes. Additionally, a lightweight dynamic upsampler, Dynamic Sample (DySample), is introduced to decrease computational complexity of the upsampling process by dynamically adjusting interpolation positions. Finally, the lightweight module Spatial Context-Aware Module (SCAM) is utilized to accurately map the connection between small targets and global features, enhancing the discrimination of small targets from the background. Experimental results demonstrate that the improved PSDS-YOLOv8 model achieves Mean Average Precision(mAP) 50 and mAP50:95 scores of 96.5% and 55.2%, which increases by 2.8% and 4.4%, while the number of parameters is reduced by 40.6% in comparison with the baseline YOLOv8 model. Compared to YOLOv5, YOLOv7, YOLOv9, YOLOv10, YOLOv11, Faster RCNN, SSD, and RetinaNet, the improved model demonstrates superior accuracy and fewer parameters, exhibiting the best overall performance. The methodology proposed in this study enhances model accuracy while concurrently reducing resource consumption and effectively addressing the issues of missed and false detections of wheat ears, thereby providing technical support and theoretical guidance for intelligent counting of wheat ears in UAV imagery.

## 1 Introduction

As a major agricultural nation, China recognizes wheat (*Triticum aestivum* L.) as not only the second largest grain crop domestically but also one of the three principal cereal crops globally, holding a significant position in agricultural production; its yield is a critical index for assessing agricultural productivity (Zhao et al., 2019; Bao et al., 2020). The density of wheat ears per unit area is a key indicator of winter wheat yield; therefore, rapid and accurate identification of wheat ears and quantification of ear density are of significant importance for predicting wheat yield, optimizing breeding strategies, and conducting plant phenotyping analysis (Xu et al., 2020; Zhang et al., 2019; Xiong et al., 2019). Traditional ear of wheat counting methods typically necessitate manual field operations, which are time-consuming, labor-intensive, and inefficient, rendering them unsuitable for large-scale implementation (Tan et al., 2020; Alkhudaydi et al., 2022). In recent years, with continuous advancements in computer vision, automated wheat ear detection techniques have begun to replace manual detection, achieving a leapfrog development from traditional image processing to machine learning and subsequently to deep learning.

In early research on wheat ear recognition, the majority of studies achieved segmentation, detection, and counting of wheat ears by analyzing their texture features and performance in a mixed color space, in conjunction with traditional image processing methods such as multi-feature fusion of color, grayscale, and texture (Fan et al., 2015; Huang et al., 2021). Zhou et al. (2018) developed a feature training model, TWSVM-Seg, which is advantageous for wheat ear recognition through feature fusion, thereby enhancing performance of wheat ear segmentation; Li et al. (2018) extracted the saturation component of the image by transforming color space and employed image preprocessing techniques, including the removal of attachments and concave point detection matching segmentation, to achieve segmentation and counting of wheat ears; Fernandez-Allego et al. (2020) eliminated low-frequency elements in the image using a Laplace frequency filter and employed a median filter to reduce high-frequency noise, and finally applied the Find Maxima Segmentation technique to accomplish segmentation and detection of wheat ear images. However, accuracy of traditional image processing techniques is constrained by the quality of images themselves. In the complex and variable field environments, factors such as lighting conditions, crop density, and occlusion significantly affect image quality, thereby diminishing accuracy of wheat ear recognition. Consequently, traditional image processing methods struggle to be effectively applied to the task of wheat ear detection across various scenarios, resulting in limited generalizability.

In contrast to traditional image processing techniques, machine learning methods exhibit greater flexibility and efficiency within the domain of image analysis. When confronted with problems such as limited data volume, low data dimensionality, and linear relationships, machine learning techniques can fully leverage algorithmic advantages, thereby significantly enhancing image processing performance. Liu et al. (2019) employed color feature clustering as a foundation to establish a direct mapping relationship between low-level features of the image and the number of wheat ears, calculating the wheat ear count by refining K-means method; Olgun et al. (2016) employed DSIFT(Dense Scale Invariant Feature Transform) for feature extraction and subsequently applied support vector machine classification algorithm to identify and detect wheat ears. However, due to machine learning model construction process, selection of target features needs to be determined by human beings, resulting in the model not effectively capturing key information in the data, leading to unstable detection results.

The rapid advancement of crop phenomics research has led to widespread application of target detection methods on the basis of deep learning in agriculture, attributed to advantages, including efficient feature extraction capabilities and adaptability to complex agricultural scenarios. These methods have significantly progressed in detecting counts of wheat ears per unit area (Bar-Hillel, 2021; Mohanty et al., 2016; Wang et al., 2021). Wang et al. (2019) combined the Fully Convolutional Network (FCN) with Harris corner detection to identify wheat in the field, enhancing detection generalizability; Guan et al. (2024) proposed the CTWheatNet model, which integrates local features with global contextual information to enhance feature representation capabilities for accurate detection of wheat ears in the field; Yang et al. (2024) introduced a lightweight density estimation network for calculating the number of wheat ears. The network is based on a ghost network for multi-scale feature extraction, combined with the FIDMT network, and adds dense upsampling modules to improve image resolution. A maximum value detection strategy is also designed to reduce background noise and interference, achieving automatic counting of wheat ears; Misra et al. (2020) integrated two innovative feature networks, the Local Patch Extraction Network (LPNet) and the Global Mask Refinement Network (GMRNet), to achieve accurate counting of pot-grown wheat plants; Gao et al. (2022) used the YOLOv3 algorithm to achieve automatic recognition of wheat ears and developed a standardized real-time estimation method for the number of wheat ears per unit area. However, the YOLOv3 model has a slower computational speed; Yang et al. (2022) developed a unit area wheat ear detection method based on improved YOLOX, which solves the problems of dense and occluded targets through Content-Aware ReAssembly Feature Extraction(CARAFE) upsampling and iAFF iterative attention feature fusion. YOLOX is based on YOLOv3 SPP and combines anchor free detection mechanism, but this model has a high missed detection rate for small targets; Gong et al. (2020) used YOLOv4, which achieved a balance between speed and accuracy, as the base network, and incorporated dual-space pyramid pooling (SPP) to improve feature learning capabilities, thereby achieving accurate detection of wheat ears; Zhao et al. (2021) improved YOLOv5 model by establishing *a priori* anchor frames and adjusting the confidence loss function of the detection layer on the basis of IoU, optimizing feature extraction for small-sized wheat ears and improving detection accuracy and precision under occlusion conditions. Yolov5 further improves the detection accuracy and speed of the model, and also has lightweight features; Meng et al. (2023) proposed the YOLOv7-MA model to accurately identify wheat ears in complex field backgrounds, enhancing the model’s ability to detect wheat ears in complex backgrounds. Both the publicly available GWHD dataset and the wheat ear dataset collected in the field can maintain good counting performance. YOLOv7 is suitable for detecting complex backgrounds and high-density targets, but the demand for computing resources also increases accordingly; Fang and Yang (2025) proposed a lightweight improvement method based on YOLOv8n. Improvements such as removing large object detection heads, replacing FPNs, and simplifying SPPF modules have reduced memory usage and increased detection speed; YOLOv8 adopts a more efficient structure, further reducing computational costs and making it suitable for edge devices and low-power devices; Guan et al. (2024) Guan significantly improves feature extraction and detection capabilities based on the YOLOv10 algorithm by introducing a bidirectional feature pyramid network (BiFPN), a separation enhanced attention module (SEAM), and a global context network (GCNet). YOLOv10 further improves performance and efficiency compared to YOLOv8.

While ground-based image analysis remains an important aspect of wheat counting research, in recent years, with maturation and widespread application of drone technology, utilization of drones for data collection offers advantages of convenience, efficiency, and extensive coverage, facilitating rapid acquisition of large-scale images of wheat ears (Araus and Cairns, 2014). However, UAV images typically exhibit lower resolution compared to ground-collected images and are characterized by small wheat ear sizes, dense distributions, and significant overlap, which can lead to issues such as occlusion, missed detections, and false detections. Additionally, the complex backgrounds of field images and morphological differences among various wheat ears further complicate detection efforts. This is particularly true in resource-constrained real-time processing scenarios, where demands for model performance are heightened. In summary, application of UAV imagery in the field of wheat ear counting still faces numerous challenges, necessitating in-depth exploration and refined research. Zhao et al. (2022) proposed the OSWSDet method, based on the YOLOv5 architecture, to address challenges posed by small, densely distributed, and heavily overlapping wheat ears in UAV images. By employing CSL (rotating target detection) to mitigate background interference, this method effectively resolves issues of occlusion and overlap. The final model achieved an average accuracy of 90.5% for wheat ear detection. Ma et al. (2022) introduced the cross-platform wheat ear counting model, EarDensityNet, which transforms wheat canopy images into ear density maps by integrating filtered pyramid blocks and extended convolution. The wheat ear count is derived by summing the pixel values of the density map. Leveraging transfer learning, the ground-based model was retrained using UAV images, resulting in the EarDensityNet_TL model. The final ground-based count error was significantly lower than the UAV-based count error, highlighting that ground resolution plays a critical role in the accuracy of UAV-based wheat spike counting.

In this study, we present a UAV wheat ear counting model, PSDS-YOLOv8, on the basis of an improved version of YOLOv8s. To tackle the challenge posed by small wheat ear sizes, we incorporate a new high-resolution P2 micro-scale detection layer while removing P5 large-scale detection layer. This adjustment improves precision by capturing high-resolution feature maps, thereby enhancing the model’s ability to recognize small targets and reducing missed detection rate of dense wheat targets. To tackle the issue of occlusion caused by background confusion in field environments, we employed spatial pyramid dilated convolution (SPD-Conv) to replace traditional subsampling methods, thereby minimizing feature loss and extracting multi-scale features related to wheat ears more efficiently, while mitigating interference from background factors such as wheat straw and leaves. To address the increase in model parameters and computational volume resulting from enhancements, we introduce lightweight dynamic upsampler, DySample, and spatial context-aware module, SCAM. These components aim to reduce computational complexity during upsampling process and enhance global correlation across space, thereby achieving model lightweighting.

## 2 Materials and methods

### 2.1 Data acquisition and processing

#### 2.1.1 Data sources

**Figure 1:**
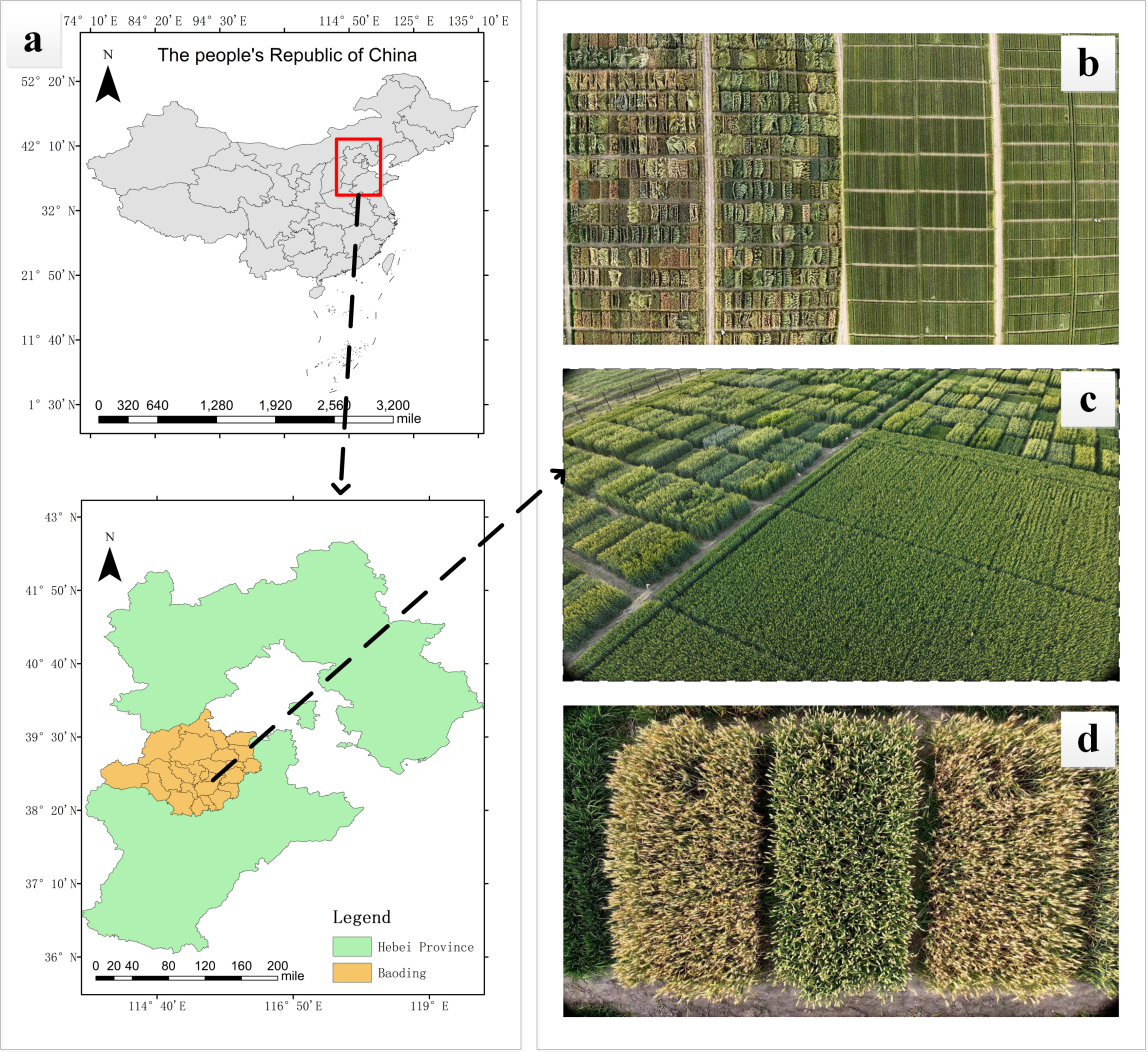
Experimental location and UAV wheat ear images: **(A)** Experimental site; **(B, C)** Actual wheat field scenarios; **(D)** UAV images of wheat ears.

The experimental site for this study is located in the experimental fields of Hebei Agricultural University in Baoding City, Hebei Province (38°48’N, 115°25’E), with geographical location depicted in **Figure 1A** and the actual wheat field scenarios illustrated in **Figures 1B, C**. Data collection was conducted from May 8, 2024, to June 11, 2024, during which various time periods and weather conditions were selected for data acquisition. A total of 17 wheat varieties were captured in images during the filling stage, the maturity stage, and the full ripe stage, effectively enhancing diversity of the dataset. The imaging equipment is the DJI Mavic 3M drone, which is equipped with a 20 megapixel camera with a focal length of 24 mm and an imaging angle of -90° to 35°. To ensure the accuracy of image acquisition, we compared the image acquisition effects at different heights. The results showed that if the drone flies at a high altitude, the image clarity will be reduced, while when flying below 3 m, the disturbance of the wheat canopy caused by airflow will interfere with image acquisition and affect image quality. Therefore, the final imaging angle of the drone lens was determined to be -90°, and 441 wheat ear images were collected by hovering at a fixed point 3m above the wheat canopy, aiming to obtain clear wheat ear images from the best perspective. An example of the wheat ear images obtained by the drone is shown in **Figure 1D**.

**Figure 2:**
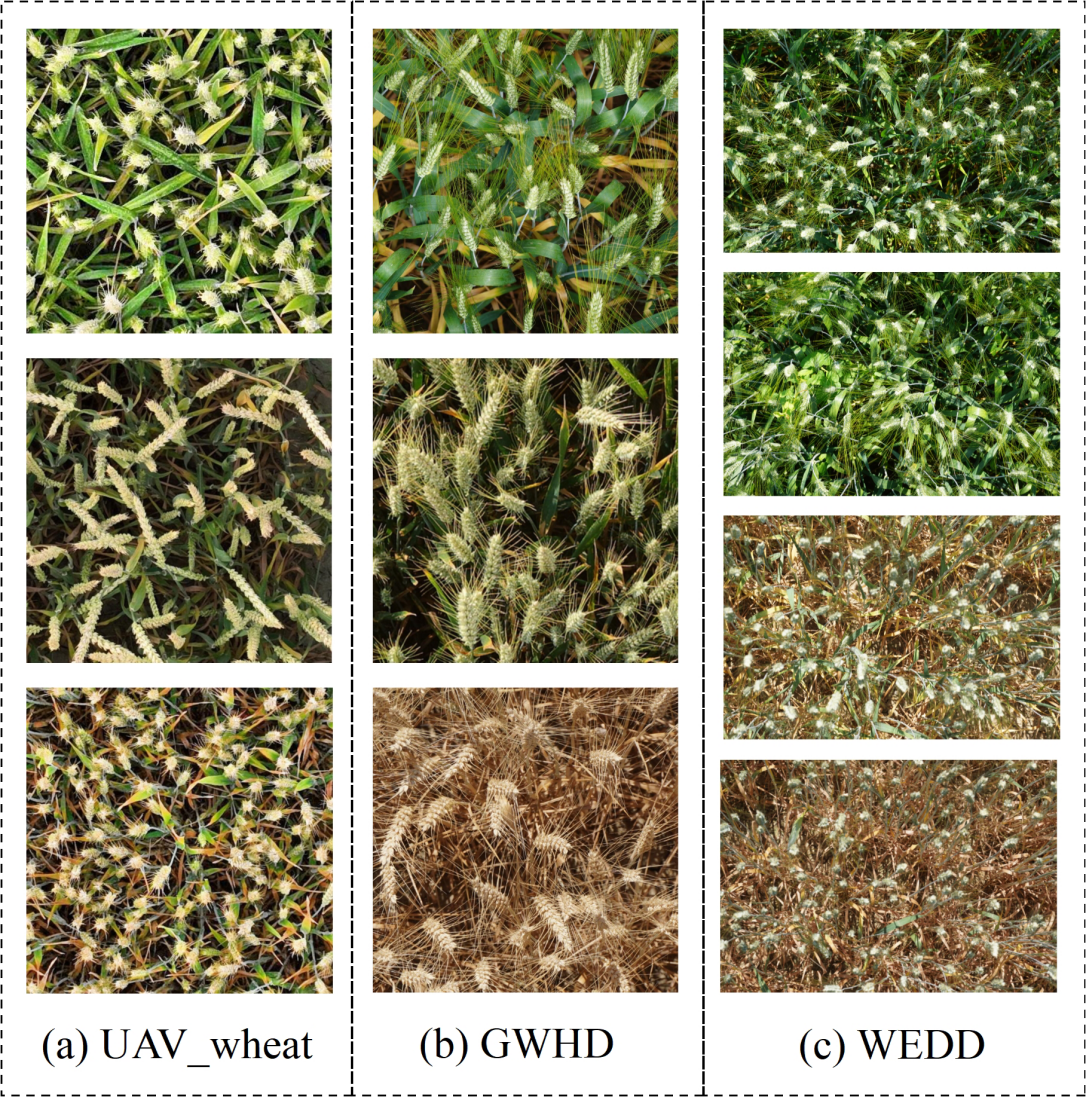
Example of wheat ear samples: **(A)** UAV wheat data image; **(B)** Global wheat head detection; **(C)** Wheat ear detection dataset.

In order to comprehensively capture the various growth conditions of wheat ears in real environments, the UAV wheat ears image database consists of wheat images under different densities, light intensities, shading levels, and other complex situations. In addition, two publicly available wheat datasets are used in this paper to enrich the composition of the data so as to validate the generalization performance of the model. The GWHD dataset contains wheat data from multiple countries and different growth stages, covering a wide range of genotypes (Madec et al., 2019); The WEDD dataset consists of high-resolution wheat sheaf images from different regions and seasons (David et al., 2020). Some sample images from the different datasets are shown in **Figure 2**, where **Figure 2A** represents a sample of wheat spikelet data captured by a drone, **Figure 2B** represents a partial sample from the GWHD dataset, and **Figure 2C** represents a sample example from the WEDD dataset, which reflects the diversity of the wheat spikelets in the present study. These diverse image data can be used to train the model more effectively, evaluate the performance of the model in different scenarios, improve the model’s ability to recognize and analyze different growth conditions of wheat, and enhance the practical applicability of the model.

#### 2.1.2 Data enhancements

**Figure 3:**
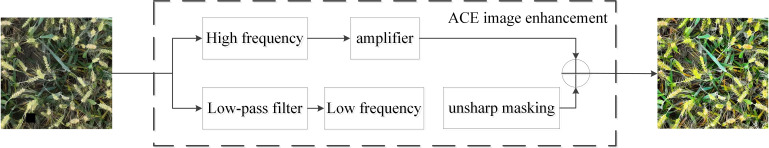
Schematic diagram of the ACE principle.

In complex field environments, variations in illumination intensity and background interference significantly affect model’s accuracy in detecting wheat ears. To mitigate the impacts of uneven lighting and intricate backgrounds on image quality, this study employs the Adaptive Contrast Enhancement (ACE) algorithm (Mahmood et al., 2019) to enhance brightness and contrast of wheat ear images acquired by UAV. This algorithm utilizes a local enhancement strategy, dividing the image into low-frequency and high-frequency components. The low-frequency component is extracted through mean filtering, and the high-frequency component, which captures detail features of the wheat ears, is obtained by subtracting the low-frequency component from the original image. An enhancement factor is then applied to amplify the high-frequency component, combined with an unsharp mask. Finally, the enhanced high-frequency image is integrated with the original low-frequency image to produce the final enhanced result (Deng et al., 2022), as illustrated in **Figure 3**. The ACE algorithm effectively suppresses low-frequency background interference, thereby enhancing visual distinction between wheat ears and the background, enabling the model to better extract target features. This process consists of the following two steps:

1. Let x(k,l) represent the gray value of a point in the image. The low-frequency component is obtained using the local averaging method. The mean value of the window of size (2n+1) × (2n+1) of the image $m_{x}\left(i,j\right)\;$ can be calculated by Equation 1, where, (i,j)  are the coordinates of the center point of the local region, and n is taken as an integer.

(1) $$m_{x}\left(i,j\right)=\frac{1}{{\left(2n+1\right)}^{2}}\sum_{k=i-n}^{i+n}\sum_{l=j-n}^{n}x\left(k,l\right)$$

The average variance within the template can be ascertained using Equation 2. $\sigma_{x}\left(i,j\right)$ is the standard deviation of a window of size (2 n + 1) × (2 n + 1).


(2) $$\sigma_{x}^{2}\left(i,j\right)=\sum_{k=i-n}^{i+n}\sum_{l=j-n}^{j+n}{\left[x\left(k,l\right)-m_{x}\left(i,j\right)\right]}^{2}$$

2. To enhance the high-frequency component, the standard deviation serves as the gain value, while the mean is approximated to represent the background, correlating with high-frequency details. The enhancement of the high-frequency component is determined by Equation 3, where D is a constant representing the gain function.

To enhance the high-frequency component, standard deviation serves as the gain value, with the mean $m_{x}$ approximated to represent background, while $\left[x\left(k,l\right)-m_{x}\left(i,j\right)\right]\;$ corresponds to high-frequency details. The gain product for the high-frequency component is calculated using the following Equation 3, where D is a constant, f(i,j) is the enhanced gain value, and the gain function is given $\sigma_{x}\left(i,j\right)$


(3) $$f\left(i,j\right)=m_{x}\left(i,j\right)+\frac{D}{\sigma_{x}\left(i,j\right)}\left[x\left(k,l\right)-m_{x}\left(i,j\right)\right]$$

The enhancement effects achieved through application of the adaptive contrast enhancement algorithm to wheat ear images (**Figure 3**). This algorithm significantly improves distinction between the target wheat ears and the background, reduces the impact of illumination on image quality, and enhances the model’s ability to capture detailed features of the wheat ears, thereby enabling more accurate recognition.

#### 2.1.3 Dataset construction

To reduce data processing time and meet the demand for data diversity in subsequent model training, 441 drone wheat images with original resolutions of 5280 pixels × 3956 pixels were cropped into 91, 200, and 150 images with 750 pixels × 750 pixels, 600 pixels × 600 pixels, and 450 pixels × 450 pixels, respectively. Then, the wheat ears in the images were labeled using the labeling tool LabelImg. After image rotation, the 441 images with different resolutions were enlarged by three times to 1323 images. The training set, validation set, and test set were constructed in an 8:1:1 ratio to complete the drone wheat dataset. Afterwards, 2200 and 200 clear wheat images were selected from two public datasets, GWHD and WEDD, respectively. The dataset details for this study is shown in **Table 1**.

**Table 1:** Dataset details for this study.

Data set	Number of images	Training set	validation set	Test set	Total number of wheat ears
UAV_wheat	1323	1058	132	133	132495
GWHD	2200	1760	220	220	118494
WEDD	200	160	20	20	29621

### 2.2 Baseline model YOLOv8

YOLOv8 is a target detection methodology released as open-source by Ultralytics on January 10, 2023. This architecture builds upon the foundation established by YOLOv5 and supports a diverse array of complex tasks, including instance segmentation, keypoint detection, object detection, and classification. The network architecture comprises three primary components: Backbone, Neck, and Head (Terven et al., 2023).

The Backbone primarily processes input images and generates feature maps required for object detection (Redmon and Farhadi, 2018). Drawing inspiration from the CSP module concept in YOLOv5, C3 module has been replaced with a more lightweight C2f module. The Neck utilizes an FPN-PANet structure to enhance capabilities of feature representation (Lin et al., 2017). In the Head, a decoupled head approach is implemented, effectively separating classification and detection heads while eliminating the object branch, thereby enabling the model to learn category and spatial features with greater efficiency (Ge, 2021). Meanwhile, YOLOv8 realizes anchor free detection, which simplifies the model training process.

Furthermore, YOLOv8 refines loss function and training strategy by employing a Task-Aligned Assigner for matching of positive and negative samples, thereby addressing class imbalance scenarios and enhancing the model’s detection capabilities for minority class samples. The selected loss functions include classification loss (VFL Loss) and regression loss (CIOU Loss + DFL), thereby improving the accuracy of bounding box predictions amidst substantial variations in object shapes and sizes. In terms of training strategy, YOLOv8 disables Mosaic augmentation in later stages of training to further enhance model accuracy (Wei et al., 2021).

### 2.3 Improved YOLOv8 model

**Figure 4:**
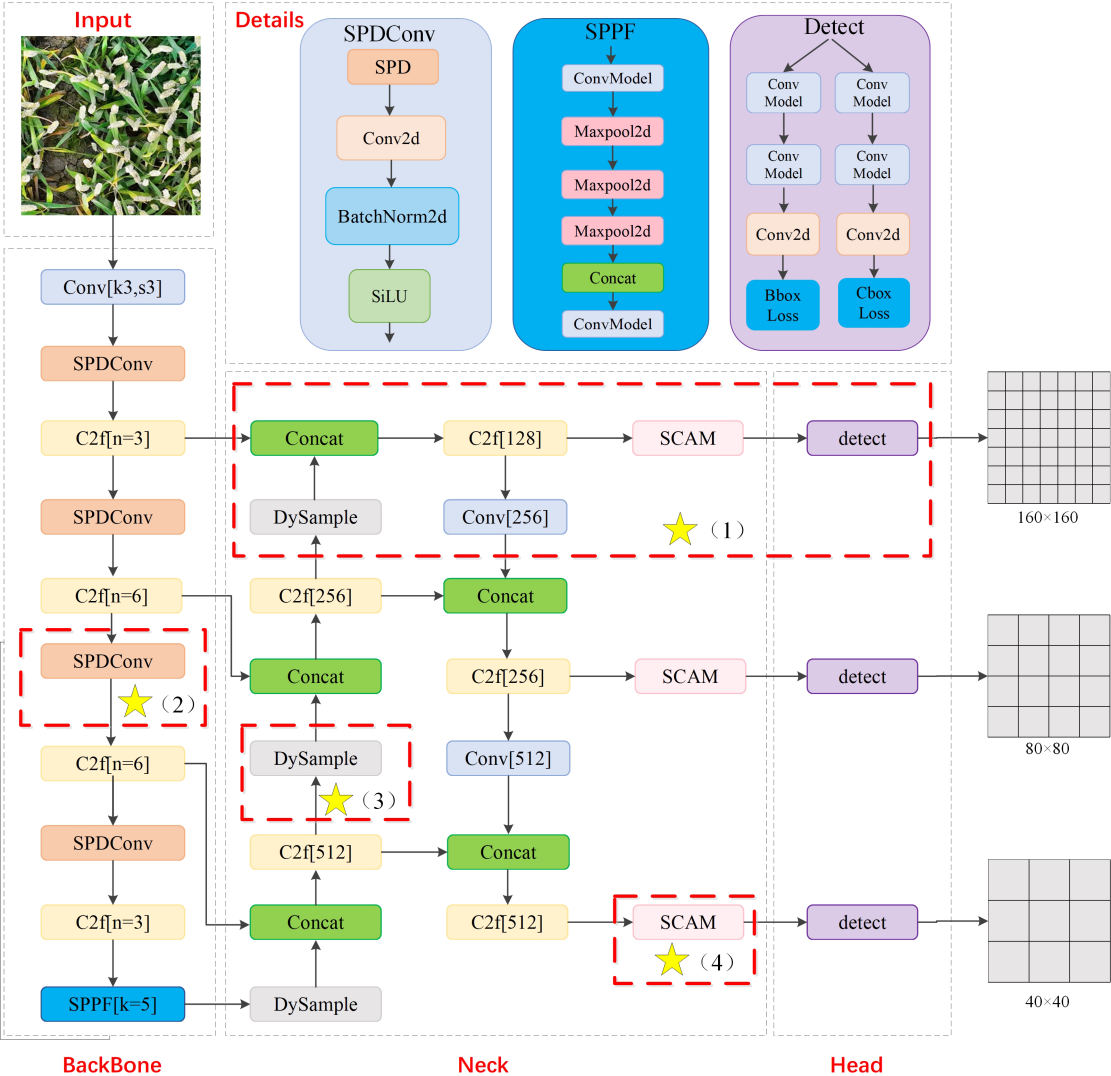
PSDS-YOLOv8 model structure. Yellow stars represent improved modules, including P2 microscale detection layer, SPD-Conv, DySample, and SCAM.

To enhance performance of wheat ear detection in UAV images, this paper presents a lightweight and efficient wheat sheaf detection model PSDS-YOLOv8 based on improved YOLOv8s. The architecture of the improved model is illustrated in **Figure 4**, where key improvement points are indicated by asterisks within the red dashed box, specifically encompassing the following four aspects of improvement:

1. To address the challenge of small target sizes of wheat ears in UAV images, a high-resolution P2 microscale detection layer has been incorporated. This layer captures more underlying feature details, thereby facilitating more effective identification of small wheat ear targets, while P5 large-scale detection layer has been eliminated to further reduce computational burden on the model.
2. In backbone feature extraction network, SPD-Conv convolution module for processing low-resolution images and small targets is introduced to replace traditional subsampling operation, helping to increase depth of feature map, enhance feature extraction capability of the model, focus its attention on dense small targets, and reduce interference from background information.
3. The upsampling method of the original model is replaced with lightweight dynamic upsampler DySample, which enhances clarity of feature edges and optimizes feature details, avoids reliance on high-resolution feature maps, and reduces computational complexity of the upsampling process, making it more suitable for real-time and resource-constrained applications.
4. Given that feature maps already provide a satisfactory representation of small-scale target features, a lightweight spatial context-aware module SCAM is adopted on this basis, aiming to construct a global contextual relationship to strengthen the association between small targets and global features, enhance differentiation between small targets and background while effectively reducing model’s computational complexity, thereby further improving detection performance.

#### 2.3.1 Enhanced microscale detection layer

YOLOv8 performs detection at three scales, subsampling the original image to 1/32, 1/16, and 1/8 of its size to accommodate detection needs of targets of different sizes. In UAV-captured wheat ear image dataset, due to small size and dense distribution of wheat ears, there are tiny targets smaller than 10 pixels. After multiple subsampling operations, these targets occupy only 1-2 pixels in feature map, resulting in severe feature information loss and an increased risk of missed detections. Additionally, baseline model’s large-scale detection layer shows no significant advantage in detecting dense wheat ears and has a longer training time. Therefore, this study removes P5 large-scale detection layer sized at 20 × 20 to reduce computational load and improve inference speed, while adding P2 microscale detection layer sized at 160 × 160, enabling the model to focus more on small wheat ear targets.

**Figure 5:**
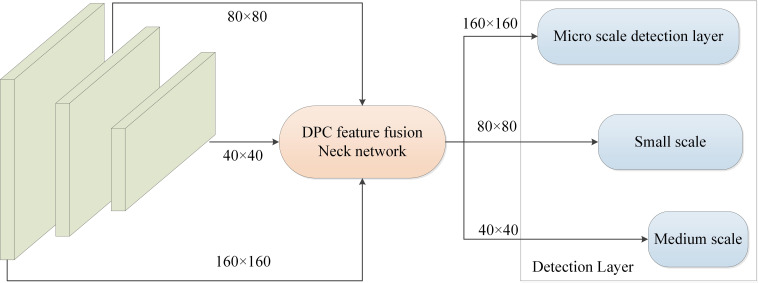
Optimization of object detection layer.

The structure of the improved detection layer is illustrated in **Figure 5**. First, 80 × 80 scale feature map generated by the second layer of the backbone network is stacked with the upsampled feature layer from the Neck. Subsequently, through DPC and upsampling processes, representation capability of small target information within feature map is enhanced, which is then concatenated with 160 × 160 feature map output from the first layer of the backbone network. The microscale detection layer maximally preserves pixel information of small wheat ears, facilitating transmission of small target features through subsampling path of detection layer. This enables the model to capture small target features within deeper layers of network, effectively reducing instances of false positives and missed detections for wheat targets of varying sizes and enhancing model’s detection capability for small wheat ear targets in aerial images.

#### 2.3.2 Spatial pyramid dilated convolution

YOLOv8 employs traditional strided convolutions and pooling layers for subsampling, which can result in loss of fine-grained information. In the context of high-resolution and large-scale object detection, loss of information has limited impact on model performance due to presence of sufficient redundant information. However, in detection tasks involving low-resolution images or smaller targets, absence of fine-grained information undermines the model’s ability to learn effective features, thereby significantly reducing detection accuracy.

**Figure 6:**
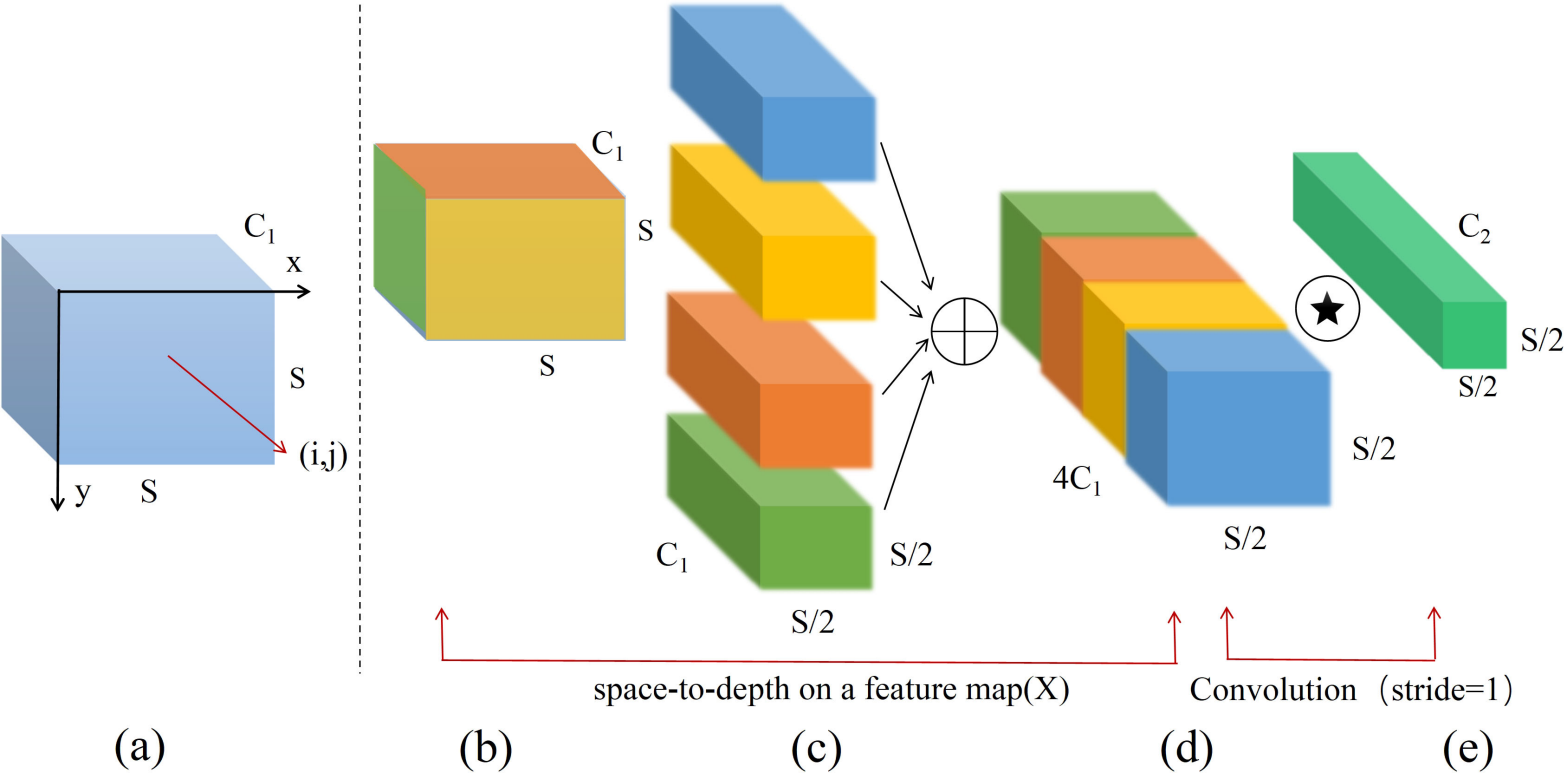
Processing of feature naps by SPD-Conv module.

Raja et al. introduced a novel convolutional neural network in 2022 known as SPD-Conv (Sunkara and Luo, 2022), consisting of a space-to-depth layer and a non-strided convolution layer. Assuming the input feature map size is S × S × C1, as illustrated in **Figure 6A**, non-strided convolution is employed to extract feature information from input feature map in greater detail while maintaining intermediate feature map dimensions at S × S × C1, as depicted in **Figure 6B**. Subsequently, the SPD layer performs subsampling operation, as shown in **Figure 6C**. This process slices the input feature map of size S × S × C1 according to a specified depth factor to obtain four sub-feature maps, each with dimensions of (S/2) × (S/2) × C1. The module then rearranges the pixels within each group of feature maps into depth dimension of a new tensor, concatenating the four sub-maps along the channel dimension to form final output feature map with dimensions of (S/2) × (S/2) × 4C1, as shown in **Figure 6D**. After processing through the space-to-depth layer, feature map is further processed by the non-strided convolution layer to yield a feature map of size S/2 × S/2 × C2, as illustrated in **Figure 6E**. The output is then convolved via Conv layer on each pixel or feature map, thereby preventing excessive subsampling loss and effectively retaining critical spatial feature information.

The SPD-Conv module transforms the spatial information of the image into depth information, reducing spatial dimension size while retaining effective information in the spatial dimension. This enables convolutional neural networks (CNNs) to learn image features more effectively and addresses issues of low image resolution and small target detection in field scenarios. In the densely UAV wheat ear dataset, proportion of small targets is significant. If traditional subsampling methods are employed, the defects of small targets are easily lost during the processes of strided convolution and pooling. Therefore, this paper introduces SPD-Conv structure to replace traditional subsampling methods, thereby reducing information loss to a certain extent and enhancing model’s capability to process small targets and low-resolution images.

#### 2.3.3 Dynamic upsampler dySample

DySample (Dynamic Sample) is an ultra-lightweight and efficient dynamic upsampling operator proposed by Mango team (Liu et al., 2023). Compared to static upsampling operators (such as bilinear interpolation and nearest neighbor interpolation), DySample better preserves feature details and fully utilizes rich semantic information within the feature map, exhibiting fewer limitations. DySample reconstructs the upsampling process through point sampling, dynamically selecting sampling points directly on feature map rather than generating dynamic convolution kernels to reorganize the feature map, significantly reducing computational complexity. In contrast to traditional kernel-based dynamic upsampling methods, such as CARAFE (Wang et al., 2020), FADE (Lu et al., 2022a) and SAPA (Lu et al., 2022b), DySample has fewer parameters and lower computational requirements, which can decrease model’s inference time and memory usage, while also reducing computational costs. Therefore, this paper proposes integrating the DySample module into YOLOv8 to replace traditional static upsampling, aiding in the reduction of computational complexity during upsampling process and ensuring the efficiency and effectiveness of the upsampling procedure.

**Figure 7:**
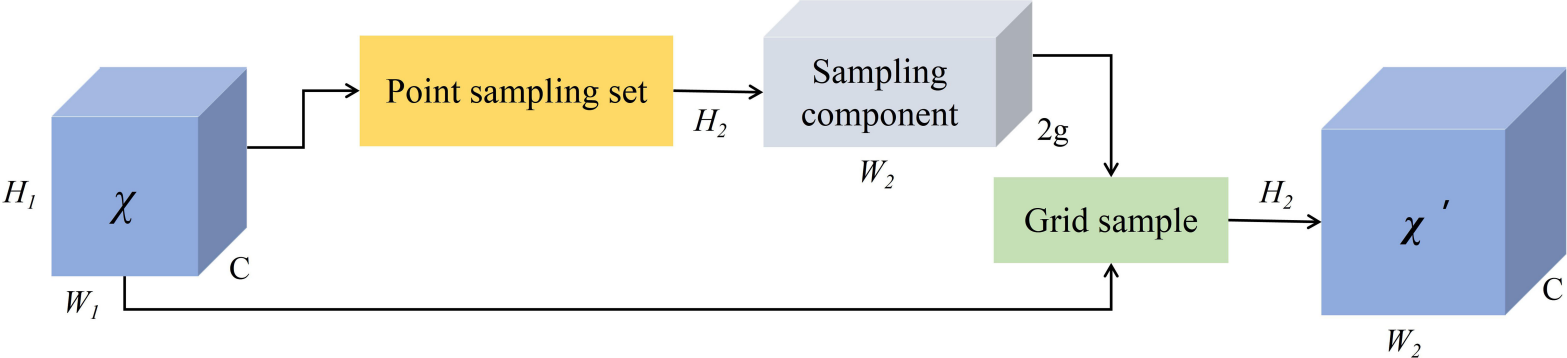
Dynamic upsampling of dySample module.


The specific workflow of DySample is illustrated in **Figure 7**, given a feature mapping of size C × H1 × W1 and a point sampling set of size 2g × H2 × W2, where 2g in the first dimension represents x and y coordinates. The feature mapping is resampled to size C × H2 × W2 by the grid_sample function using the positions in the point sampling set as shown in Equation 4.

(4) χ＇= grid_sample(χ,δ)

**Figure 8:**
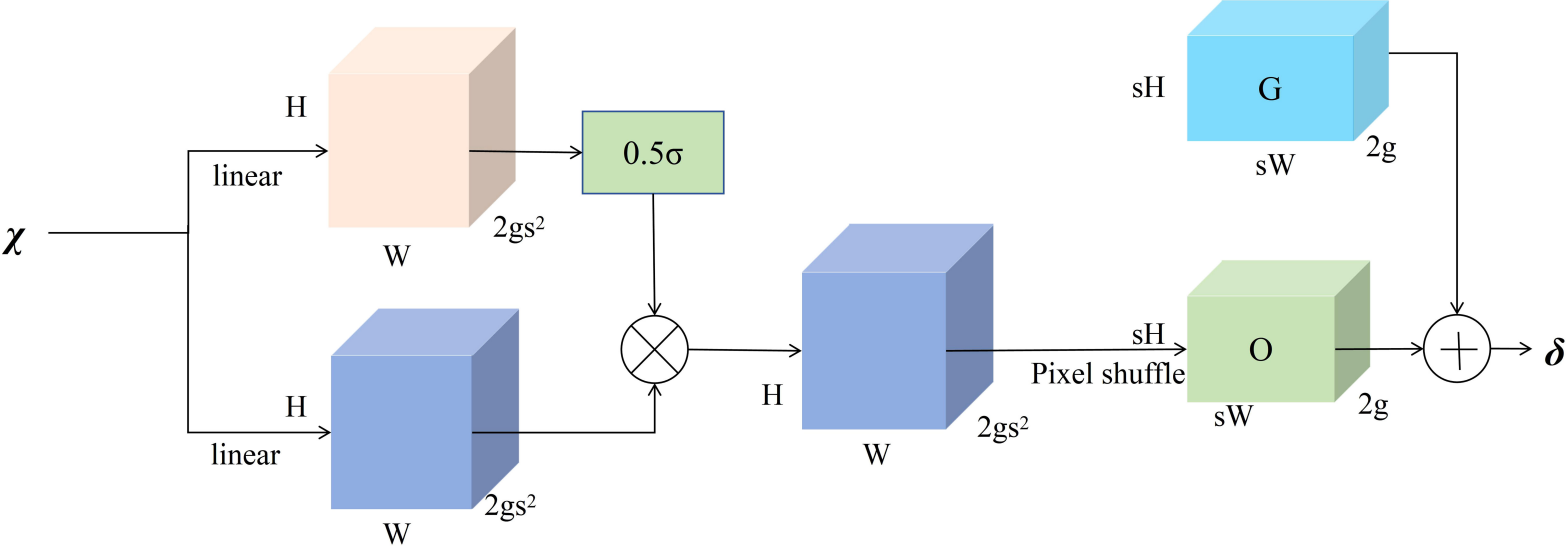
Point sampling based on dynamic range factor.


The generation of point sampling set based on the dynamic range factor is shown in **Figure 8**. First, the DySample module receives input low-resolution feature maps and employs a linear layer to generate offsets for each point. Given upsampling scale factor s and the feature mapping χ of low-resolution feature map of size C × H × W, a linear layer with input and output channel numbers C and 2gs^2^ respectively generates offsets O of size 2gs^2^ × H × W, as shown in Equation 5:

(5) O=linear(χ)

It is then reshaped into a high-resolution raw sampling network G of size 2g × sH × sW by pixel shuffle, and the generated offset O is added to the raw sampling network G to form the final set of point samples δ as shown in Equation 6:


(6) δ=O+G

Through the aforementioned steps, DySample dynamically determines generation and positional adjustment of sampling points based on content of input feature map, achieving dynamic upsampling from low-resolution feature maps to high-resolution feature maps.

#### 2.3.4 Lightweight construction SCAM

**Figure 9:**
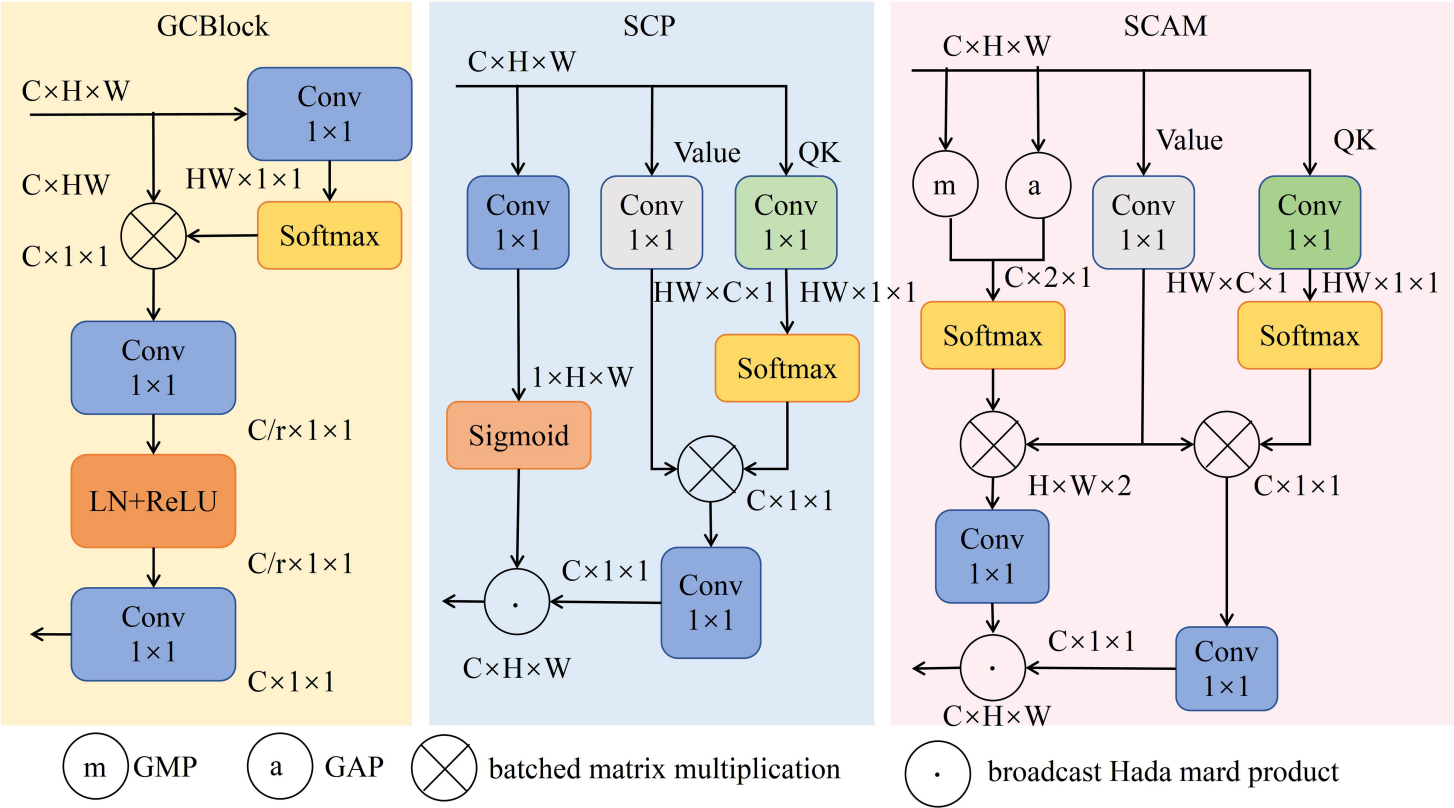
Structure of GCBlock, SCP and SCAM.

The Spatial Context-Aware Module (SCAM) comprises three distinct branches, as depicted in **Figure 9**. First branch extracts global context information from feature map through Global Average Pooling (GAP) and Global Max Pooling (GMP) operations. Second branch employs a 1×1 convolutional layer to generate linear transformation result, denoted as “value,” from feature map. The third branch, referred to as QK, utilizes a 1×1 convolution to simplify multiplication of query and key. Consequently, first and third branches are respectively multiplied by second branch, generating two branches that represent contextual information across channels and spatial dimensions. The outputs of these two branches are combined using a broadcast Hadamard product to form output of SCAM. SCAM effectively enhances global contextual awareness of feature map, leveraging global information to strengthen spatial and channel relationships within feature map, facilitating cross-channel and spatial feature fusion, thereby improving model’s accuracy in object recognition.

Following enhancement of detection layer and integration of SPD-Conv and DySample modules, representation of small target features within feature map has been significantly enhanced. Building upon this, introduction of SCAM structure strengthens connection between small targets and global features by constructing global contextual relationships (Zhang et al., 2024). By utilizing global feature information, SCAM enables interaction of contextual features across channels and spatial dimensions, allowing model to accurately distinguish small targets from the background. Furthermore, this module possesses lightweight characteristics, providing robust support for high-precision real-time detection of dense wheat ears.

### 2.4 Experimental environment and training parameters

The environmental configuration for this experiment is presented in **Table 2**. All models in this study will employ the same evaluation methods and parameters for comparison, facilitating the verification of their effectiveness. The parameter optimizer selected for the training process is SGD; The input size for model training is 640 × 640; the patience for early stopping is set to 100 epochs; the batch size is configured to 16; the training is conducted on a GPU; the number of worker threads for data loading is set to 4; the initial learning rate (lr0) is set to 0.01, the cosine annealing parameter (lrf) is set to 0.01, the weight decay is set to 0.0005, and the learning rate momentum (momentum) is set to 0.937. Based on multiple experiments, number of epochs is set to 200 to prevent model overfitting.

**Table 2:** Configuration parameters of the experimental environment.

Hardware	CPU: Intel(R) Core(TM) i9-13900K RAM:128GB GPU: NVIDIA GeForce RTX 3090
Environment	Windows10 64bit Python:3.8
Software	Pytorch-gpu:1.10.0 CUDA:12.2

### 2.5 Evaluation metrics

In this study, Precision (P), recall (R), mean average precision (mAP), parameters, floating-point operations (FLOPs), mean absolute error (MAE), mean square error (MSE), Coefficient of Determination (R^2^) and Accuracy are chosen as evaluation metrics.

The mean average precision is related to the precision (P) and recall (R), calculated using the following Equations, where TP represents number of true positive detections, FP denotes the number of false positive detections, and FN indicates the number of false negatives.


(7) $$P=\frac{TP}{TP+FP}$$

(8) $$R=\frac{TP}{TP+FN}$$

Average Precision (AP) is defined as area under the Precision-Recall (P-R) curve, which is formed by the combinations of different Precision and Recall values. A larger area under the PR curve corresponds to a higher AP value, indicating improved average precision of the model and better detection performance for wheat ears. The calculation Equation is as follows:


(9) $$\text{AP}=\int_{0}^{1}\text{P}\left(\text{R}\right)\text{dR}$$

Mean Average Precision (mAP) is a commonly used metric for evaluating object detection models, with this study focusing on mAP50 and mAP50-95. mAP50 refers to average detection precision at an Intersection over Union (IoU) threshold of 0.5 across all classes in the dataset, while mAP50-95 indicates average detection precision across all classes at IoU thresholds ranging from 0.5 to 0.95. A higher mAP value signifies superior detection performance of model for dense wheat ears in UAV imagery. The calculation Equation is as follows, where m denotes the number of categories of target in the dataset.


(10) $$mAP=\frac{\sum^{}AP}{m}$$

Mean Absolute Error (MAE) is a common loss function used in regression modeling loss function that reflects the distance between the estimated and true values, where n is the number of test samples; $e_{i}$ is the number of targets in the *i*-th image estimated by the model; and $a_{i}$ is the actual number of targets from the *i*-th image that was labeled. as shown in Equation 11:


(11) $$MAE=\frac{1}{n}\sum_{i=1}^{n}\left|e_{i}-a_{i}\right|$$

Mean Squared Error (MSE) represents the stability of estimating the number of targets, The larger the MSE, the more likely it is that the estimated results exist Outliers. The definition of MSE is as follows:


(12) $$MSE=\frac{1}{n}\sum_{i=1}^{n}{\left(e_{i}+a_{i}\right)}^{2}$$

Coefficient of Determination (R2) is used to measure the fit between the model and predicted data. The value range is 0 to 1, with values close to 1 indicating good model fit and values close to 0 indicating poor fit. The calculation formula is as follows, where ${\text{SS}}_{\text{residual}}$ is the sum of squared residuals, and ${\text{SS}}_{\text{total}}$ is the sum of squared residuals.


(13) $$R^{2}=1-\frac{SS_{residual}}{SS_{total}}$$

Accuracy is one of the most intuitive performance metrics, measuring the percentage of predictions that the model gets right.


(14) $$Accuracy=\frac{TP+TN}{TP+TN+FP+FN}$$

## 3 Results

### 3.1 The impact of data augmentation on detection results

Considering that wheat images collected in field environments can be adversely affected by uneven illumination, this study employs Adaptive Contrast Enhancement (ACE) algorithm to enhance the images. By comparing experimental results of training the PSDS-YOLOv8 model on both ACE-processed and unprocessed images, the study aims to verify the impact of image enhancement on detection outcomes, as presented in **Table 3**. The mAP50 of the original model on unenhanced dataset is 93.7%, while the mAP50 on the enhanced dataset is 94.6%, thereby confirming the necessity and effectiveness of data augmentation.

**Table 3:** Comparison of data enhancement effects.

Data processing	P (%)	R (%)	mAP50 (%)
Unenhanced	87.5	89.4	93.7
Enhanced	88.9	90.2	94.6

### 3.2 Comparison of ablation experiment results

To validate the effectiveness of proposed method, ablation experiments were conducted on the PSDS-YOLOv8 model. Results of ablation experiments, as presented in **Table 4**, indicate that after incorporating P2 microscale detection layer and removing large-scale detection layer, model achieves a reduction in the number of parameters while effectively enhancing detection accuracy. Specifically, mAP50 and mAP50:95 metrics improve by 1.3% and 3.9%, respectively, compared to baseline model, confirming its efficacy in capturing small targets. Following the addition of the SPD-Conv subsampling structure within network’s backbone, mAP50 and mAP50:95 metrics increase by 1.5% and 4.2%, respectively, further reinforcing the model’s detection capabilities in complex field scenarios. After integrating lightweight dynamic upsampler DySample in the neck of the network, the model’s adaptability to targets of varying scales and shapes is enhanced by leveraging the rich semantic information in feature map, while simultaneously simplifying computational complexity of upsampling process. This results in improvements of 1.5% and 4.3% in mAP50 and mAP50:95, respectively, along with a reduction in both model parameters and computational load. Additionally, adoption of the lightweight SCAM structure enables the model to decrease the number of parameters and computational volume, effectively enhancing detection accuracy. The experimental results demonstrate that when these four methods are simultaneously optimized, the model achieves optimal accuracy, with mAP50 and mAP50:95 values of 96.5% and 55.2%, respectively an increase of 2.8% and 4.4% over the baseline model. Although the floating-point computation slightly increases, the number of model parameters decreases by 40.6%, effectively balancing detection accuracy with resource consumption. Consequently, the model proposed in this paper significantly improves both the accuracy and efficiency of wheat ear detection.

**Table 4:** Results of ablation experiments.

P2	SPD-Conv	DySample	SCAM	mAP50 (%)	mAP50:95 (%)	Parameters (M)	FLOPs (G)
–	–	–	–	93.7	50.8	1.1	29.5
√	–	–	–	95.0	54.7	7.6	35.4
–	√	–	–	95.2	55.0	10.3	26.5
–	–	√	–	95.2	55.1	11.2	28.6
–	–	–	√	95.2	52.7	10.8	27.5
–	√	√	√	95.4	53.5	11.0	27.5
√	–	√	√	95.6	54.7	8.1	37.2
√	√	–	√	94.6	54.1	6.9	34.1
√	√	√	–	94.4	54.6	7.2	35.1
√	√	√	√	96.5	55.2	6.8	33.4

In addition, In order to evaluate the effectiveness of the DySample upsampling method proposed in this paper in replacing traditional upsampling modules in model feature fusion, we designed three different feature fusion strategies: introducing a lightweight cross scale feature fusion module CCFM (Cross-Scale Feature Fusion Module), using CARAFE (Content-Aware ReAssembly Feature Embedding) method for upsampling, and using a multi-level feature fusion module SDI (Selective Dual Integration) to reconstruct the feature fusion layer. The experimental results are shown in **Table 5** while keeping the model training parameters consistent. The data in **Table 5** shows that compared to traditional kernel based CARAFE upsampling methods, DySample has fewer parameters and lower computational complexity. When the model uses the Dysample upsampling method, the improved accuracy and efficiency of the model are optimal. The DySample method effectively achieves dynamic upsampling from low resolution feature maps to high-resolution feature maps, thereby improving image resolution and significantly enhancing the model’s ability to identify wheat ears.

**Table 5:** Effectiveness of different feature fusion methods for model improvement.

Different feature fusion methods	mAP50 (%)	mAP50:95 (%)	Parameters (M)	FLOPs (G)
CARAFE upsampling method	94.3	54.8	1.32	30.8
Multilevel feature fusion module SDI	93.5	54.6	1.68	29.7
Feature fusion module CCFM	92.9	53.7	1.39	29.2
DySample upsampling method	95.2	55.1	1.12	28.6

### 3.3 Comparison of performance among different detection models

**Figure 10:**
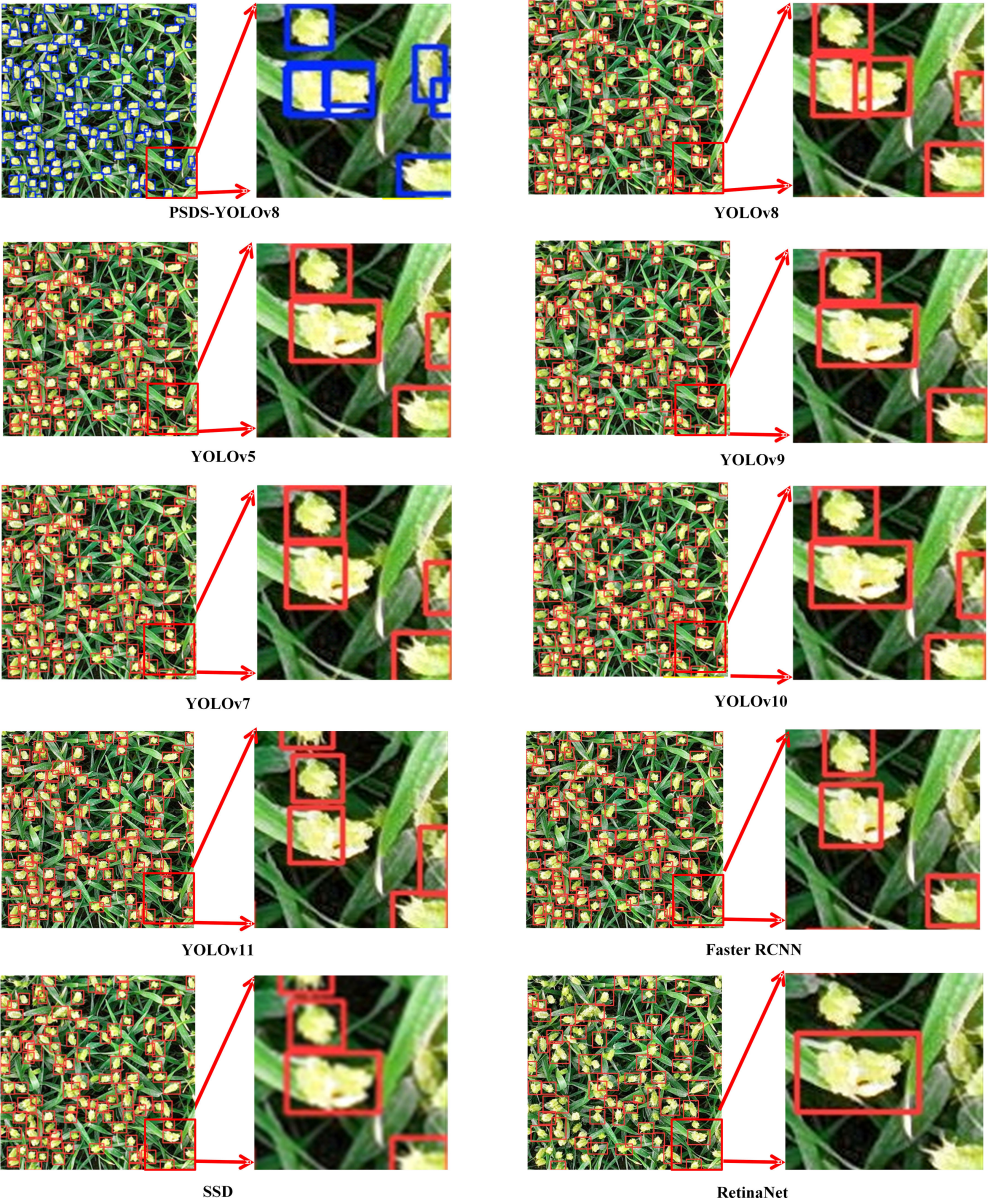
Comparison of detection effects of different models.

To further validate effectiveness of the proposed PSDS-YOLOv8 model for wheat ear detection in UAV images, this study conducts comparative experiments against mainstream object detection models, including YOLOv5, YOLOv7, YOLOv8, YOLOv9, YOLOv10, YOLOv11, Faster RCNN, SSD, and RetinaNet. As illustrated in **Figure 10**, the comparative recognition results clearly indicate that when processing small-scale targets with low resolution, the incompleteness of wheat ear features near image boundaries results in insufficient feature information for the model. Consequently, YOLOv5, YOLOv7, YOLOv9, YOLOv10, YOLOv11, Faster RCNN, SSD, and RetinaNet exhibit missed detections when identifying boundary targets or erroneously classify overlapping targets as a single entity. In contrast, YOLOv8 demonstrates relatively accurate recognition of wheat ears, although its performance in detecting overlapping targets remains suboptimal. The improved PSDS-YOLOv8 model exhibits superior recognition capabilities for wheat ears located near image edges, as well as for densely distributed occluded ears. Therefore, this model effectively mitigates interference of complex backgrounds on object detection and demonstrates improved learning outcomes for densely packed small targets, highlighting its superiority in complex field environments.

**Table 6** shows the comparison of performance indexes of different target detection models, from the comparison of performance indexes, RetinaNet has the worst results in all performance indexes, not only the recognition accuracy of the model is low, but also the number of model parameters and floating-point arithmetic is too high, which makes the model detection speed slower. the accuracy of Faster RCNN, SSD, and YOLOv9n is lower than 90%, and the number of model parameters and YOLOv5n and YOLOv11n have the smallest number of parameters and operations compared to the other models, but their recognition accuracies are low. The YOLOv7n model has a higher number of parameters and floating-point operations, but has no significant advantage over the improved PSDS-YOLOv8 model, while the YOLOv10n model has a lower number of parameters and floating-point operations, but its recognition accuracy is not optimal. The mAP50% of the improved PSDS-YOLOv8 model is 96.5%, which compares with YOLOv5n, YOLOv7n, YOLOv8s, YOLOv9n, YOLOv10n, YOLOv11n, Faster RCNN, SSD, and RetinaNet, respectively, and improves the model by 4.9%, 4.5%, 2.8%, 6.7%, 3.5%, 6.5%, 12.8%, 9.0%, 19.1%, the number of model parameters is reduced by 40.6% compared to the original YOLOv8s, and the floating-point operation volume is slightly increased, but the model has better performance in the four metrics of P, R, mAP50, and mAP50:95 by 3.9%, 3.5%, 2.8%, and 3.9%, respectively, establishing it as most effective model on the basis of comprehensive performance indicators.

**Table 6:** Effectiveness of different models in assessing indicators.

Models	Input size	mAP50 (%)	mAP50:95 (%)	Parameters (M)	FLOPs (G)
YOLOv5n	640 × 640	91.6	40.4	7.0	15.8
YOLOv7n	640 × 640	92.0	41	37.2	105.1
YOLOv8s	640 × 640	93.7	50.8	11.4	29.5
YOLOv9n	640 × 640	89.8	43.7	60.5	263.9
YOLOv10n	640 × 640	93.0	52.4	8.04	24.8
YOLOv11n	640 × 640	90.0	40.2	2.3	6.3
Faster RCNN	800 × 1333	83.7	37.9	41.8	134.4
SSD	512 × 152	87.5	40.8	26.1	91.4
RetinaNet	800 × 1333	77.4	31.5	35.4	234.6
PSDS-YOLOv8	640 × 640	96.5	55.2	6.8	33.4

### 3.4 Comparison of the effects of different wheat datasets

In order to improve the effectiveness and generalizability of the wheat counting method proposed in this study, we use the UAV-collected wheat dataset (UAV_wheat), the GWHD dataset and the WEDD dataset to train and validate our wheat detection model. **Table 7** shows the effectiveness of the model with different experimental data. Among them, the improved model works best on UAV_wheat and improves mAP50% and mAP50:95% by 2.8% and 4.4%, respectively. The results were slightly worse on the WEDD dataset, probably due to the denser distribution of wheat ears in the WEDD dataset and the higher overlap with the background information. However, the improved PSDS-YOLOv8 model achieves more than 90% precision in all three datasets, confirming the strong generalization performance of the model proposed in this study.

**Table 7:** Comparison of the effects of different wheat datasets.

Data set	Number of images	Original model		Improved model	
		mAP50 (%)	mAP50:95 (%)	mAP50 (%)	mAP50:95 (%)
UAV_wheat	1323	93.7	50.8	96.5	55.2
GWHD	2200	92.5	42.1	93.9	51.2
WEDD	200	90.4	39.8	92.3	41.6

**Figure 11:**
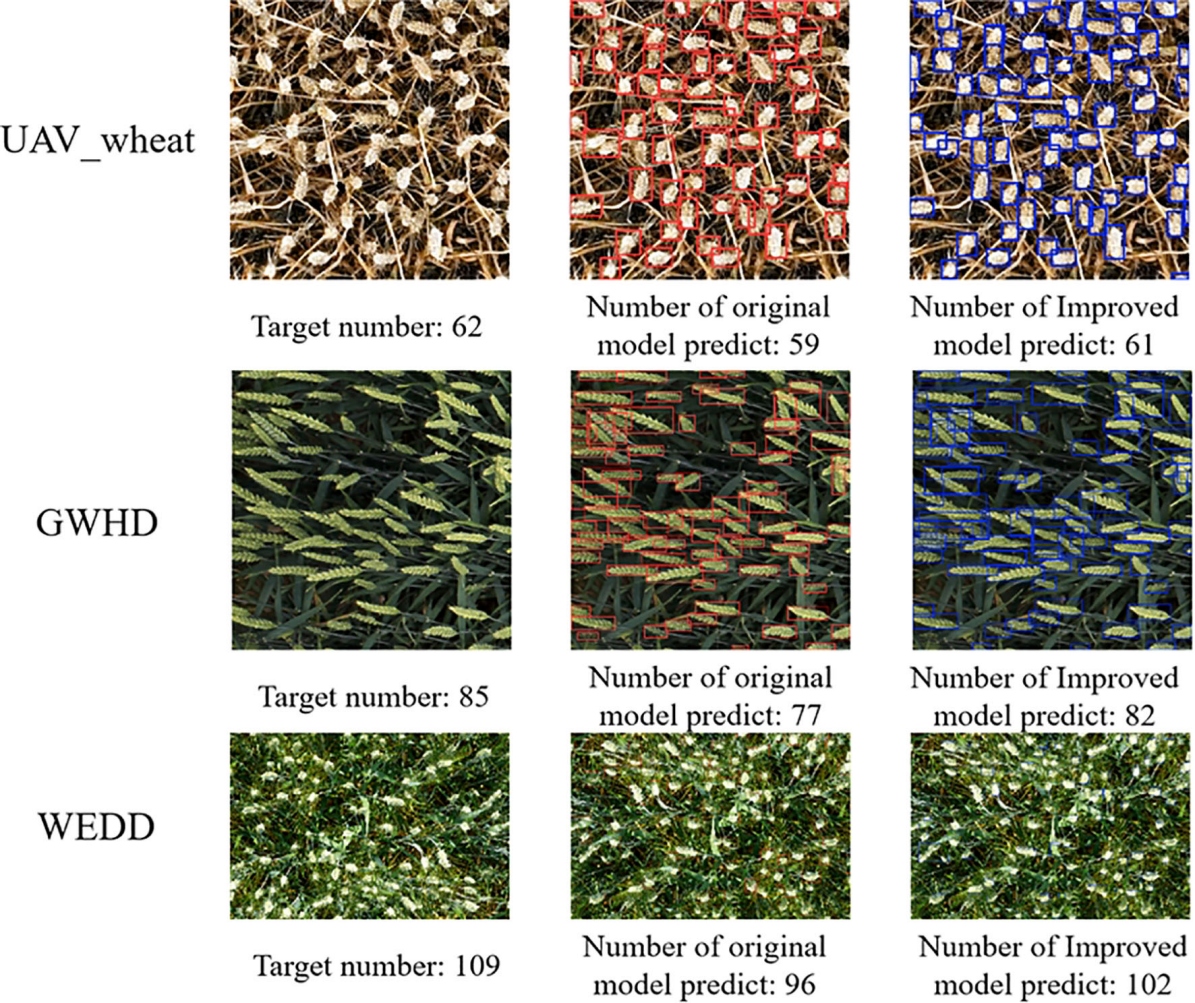
Effectiveness of wheat ear counting in different data sets.

To further demonstrate the model’s wheat counting performance, the improved PSDS-YOLOv8 model was used to test different wheat dataset samples. From the wheat counting results in **Figure 11**, it can be seen that the improved PSDS-YOLOv8 model has improved counting ability for different wheat datasets. As a result, the model has some generalization ability.

### 3.5 Comparison of effects across different growth stage

**Figure 12:**
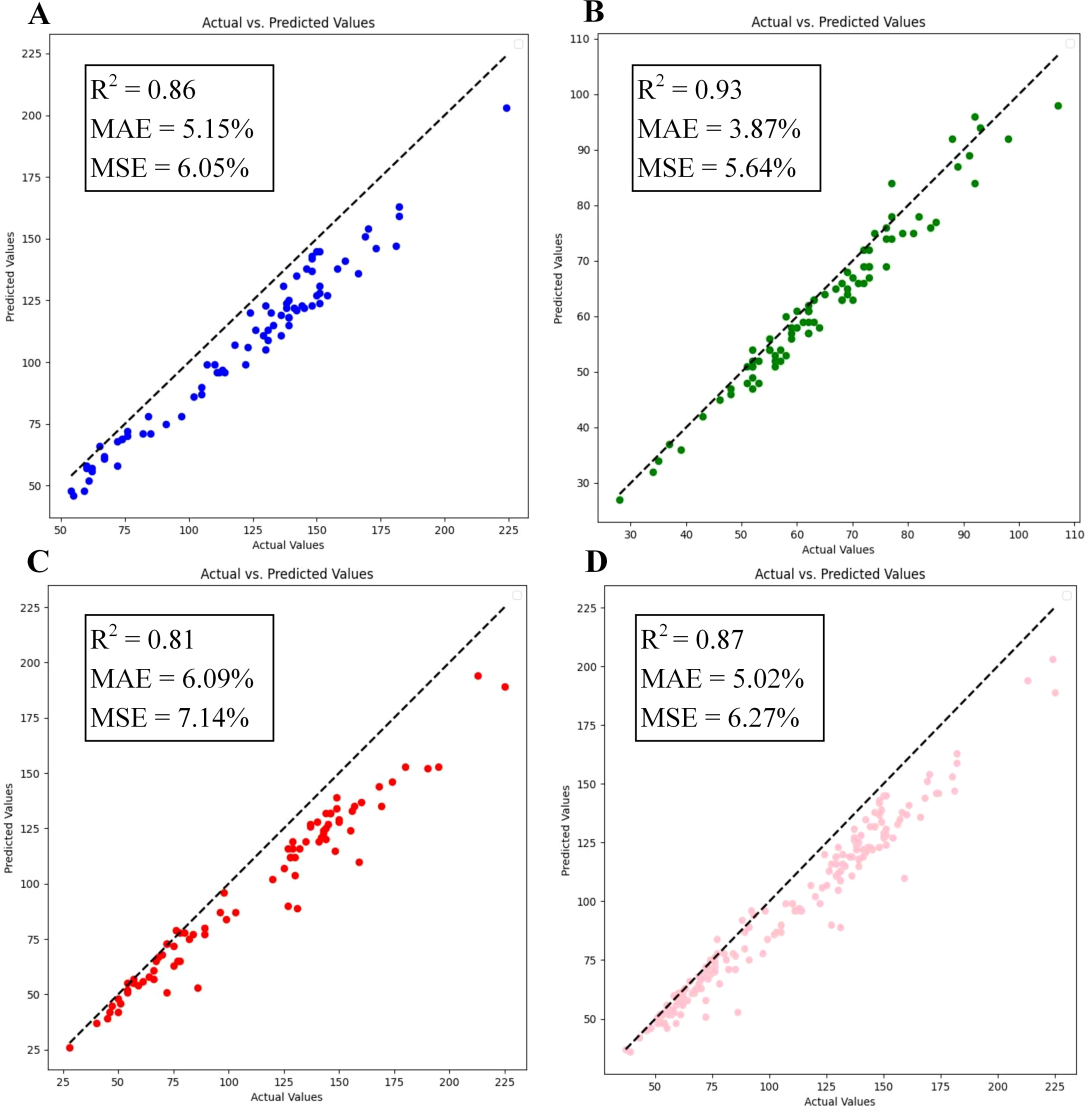
Comparison of experiments at different reproductive period of wheat: **(A)** Filling stage; **(B)** Maturity stage; **(C)** Full ripe stage; **(D)** Total.

To evaluate proposed model’s capability in detecting wheat ears at different growth stages, this study was tested using a dataset containing images of wheat at the filling stage, maturity stage and the full ripe stage. The experimental results are illustrated in **Figure 12**, where the model achieved an MAE of 5.02%, an MSE of 6.27%, and an R^2^ of 0.87 on the overall dataset. In the individual tests for different fertility stages of wheat, the model counts were able to achieve an R^2^ of 0.80 or more, and the counts were especially best on the maturity stage wheat images, where the network’s R^2^ reached 0.93, which is a high degree of fit. This confirms that the model proposed in this paper possesses strong generalization capabilities and can adapt to the various growth stages of wheat.

### 3.6 Field wheat ears instance detection

**Figure 13:**
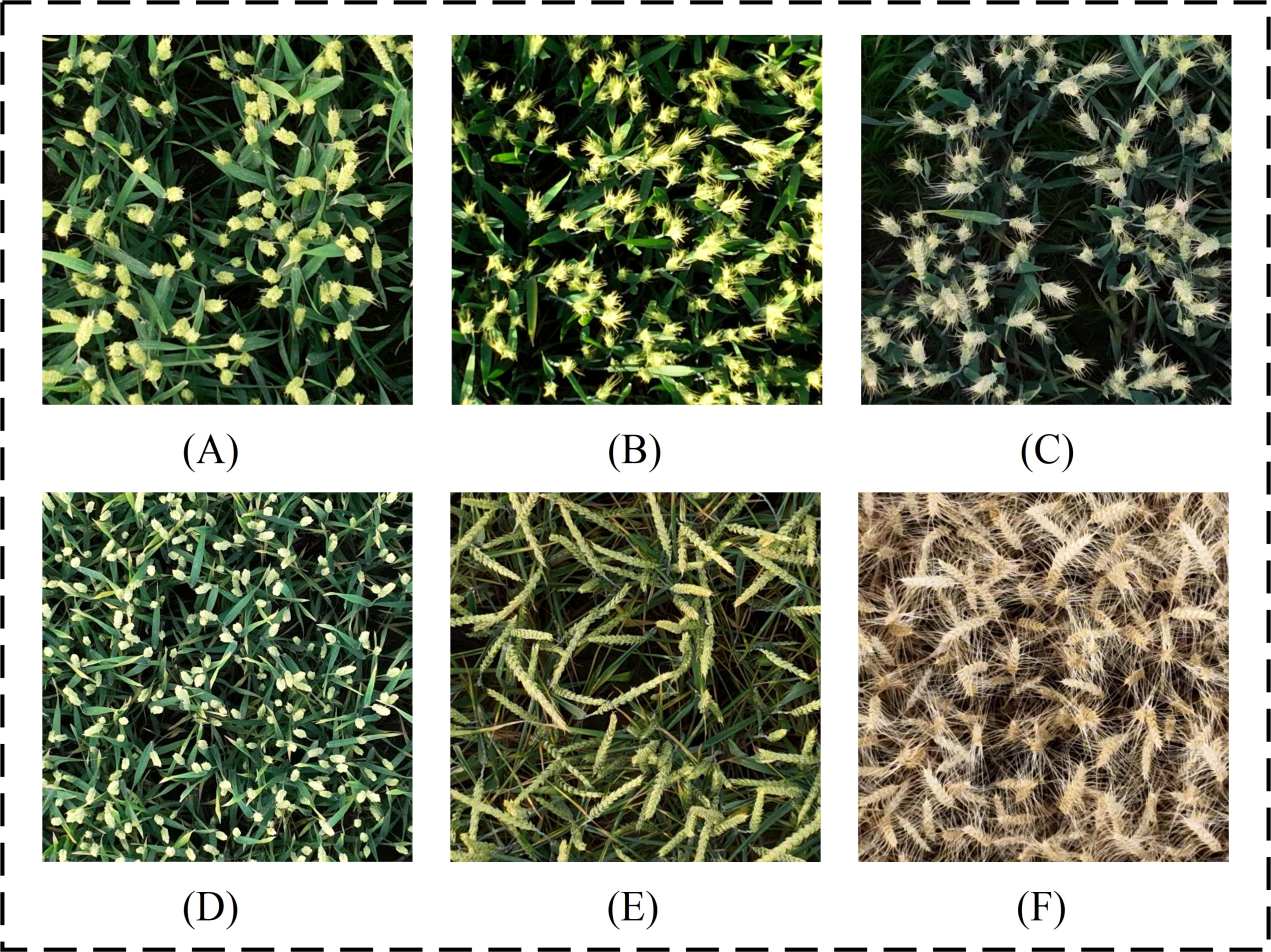
Example of wheat ear samples in the field: **(A)** Clear; **(B)** Strong light; **(C)** Darkness; **(D)** Dense; **(E)** Slight obstruction; **(F)** Serious obstruction.

This study evaluates detection performance of the PSDS-YOLOv8 model for wheat ear images across diverse wheat field scenarios, including complex conditions such as clear state, intense illumination, shaded environments, dense distributions, and overlapping occlusions, to validate its performance improvements (**Figure 13**).

**Table 8** records the model before and after the improvement is recognizing the wheat images of different scenes. In instances where wheat ear images exhibit high clarity, the original model experiences leakage and misdetections during the recognition process, whereas the PSDS-YOLOv8 maintains superior recognition accuracy; Under highlight conditions induced by intense illumination, the original model experiences significant missed detection due to effects of lighting, whereas the PSDS-YOLOv8 model substantially reduces the missed detection rate through enhanced feature extraction and improved background interference suppression; In shaded environments, both original and improved models demonstrate good detection capabilities, with no significant differences observed; In scenarios with densely packed wheat ears, the PSDS-YOLOv8 model effectively reduces missed detections due to occlusion; In cases of both slight and severe occlusion, the enhanced PSDS-YOLOv8 model significantly improves the recognition capability of wheat ears, yielding a substantially higher detection count compared to the original model. Thus, the PSDS-YOLOv8 model exhibits strong wheat ear detection capabilities across various complex field scenarios, meeting demands for adaptability in real agricultural production.

**Table 8:** Example of wheat ear detection in the field effectiveness.

Various scenarios	Clear	Strong light	Darkness	Dense	Slight obstruction	Serious obstruction
Original model count accuracy (%)	92.0	71.1	92	80.4	57.0	57.3
Improved model counts accuracy (%)	98.3	90.1	98.0	97.0	96.3	92.0

## 4 Discussion

### 4.1 Advantages of ACE algorithm

**Figure 14:**
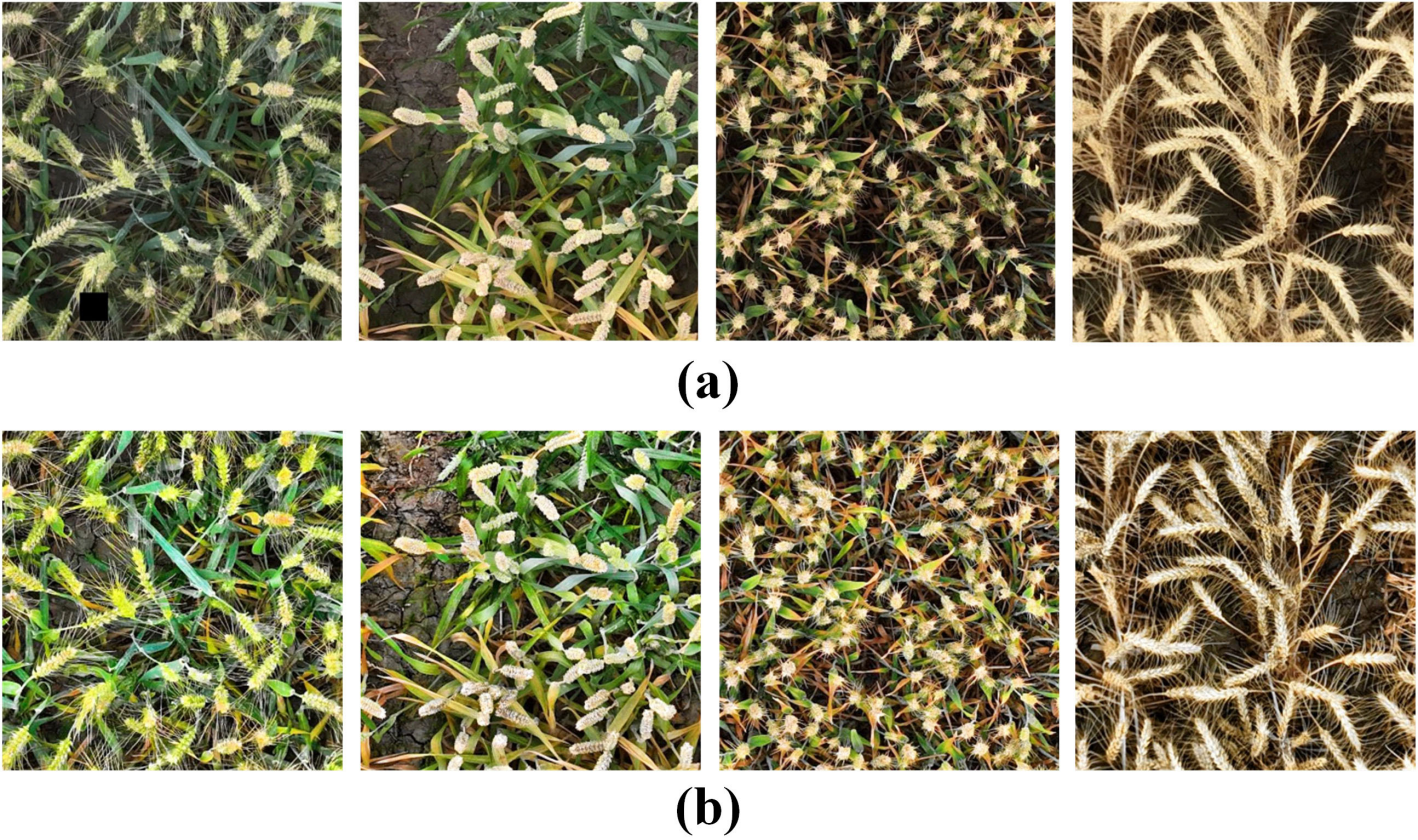
Original images and the images enhanced using the ACE algorithm: **(A)** Original image; **(B)** Images enhanced using the ACE algorithm.

In this study, Adaptive Contrast Enhancement (ACE) algorithm was employed to optimize the quality of the UAV aerial wheat dataset (**Figure 14**). The results indicate that ACE method effectively enhances the contrast between the target and the background while mitigating the impact of illumination on image analysis. Furthermore, the algorithm demonstrates significant effects in enhancing the performance of target detection models (**Table 3**). Cheng et al. (2024) confirmed the ACE algorithm’s capability to enhance visual quality while preserving essential details through comparisons of various image enhancement techniques, thereby playing a crucial role in improving accuracy and reliability of image analysis. Consequently, ACE algorithm serves as an effective tool for optimizing image quality.

### 4.2 Potentials and limitations of the work

Existing research indicates that models trained on a single sample dataset experience a significant decline in detection accuracy when applied to other wheat ear datasets, highlighting certain limitations (Dandrifosse et al., 2022). To address the issue of insufficient generalization performance, this study enhances model’s generalization capability by increasing the diversity of training samples, with a total of three different wheat datasets containing wheat ears of different varieties, growth stages and morphologies (**Figure 2**). Factors such as small wheat ear sizes and occlusion limit the feature information obtainable by the model, thereby posing challenges to wheat ear detection (Almoujahed et al., 2022), while overlap and occlusion among dense wheat ears represent the primary difficulties in this detection task. The OSWSDET method proposed by Zhao et al. (2022) effectively fuses lower spatial features with deeper semantic features through the application of a microscale detection layer, thereby providing a more accurate representation of the location and size of wheat ears. This approach is particularly effective for detecting small and densely packed wheat ears in UAV images. Yu et al. (2024) introduced SPD-Conv structure, which significantly enhances model’s ability to detect small targets, as demonstrated by increases of 2.3% and 3.4% in mAP50 and mAP50:95, respectively. This study adds a new P2 microscale detection layer to the YOLOv8 baseline model and incorporates a spatial depth-transformed convolutional SPD-Conv suitable for small target detection, thereby improving detection accuracy of tiny wheat targets in UAV images. However, high-precision recognition necessitates deeper model structure, which inevitably involves an increased number of parameters. Wu et al. (2024) employed ultra-lightweight upsampling operator DySample to reduce network’s parameter count, resulting in a 0.76% improvement in model detection speed. Yang et al. (2024) proposed a lightweight wheat ear detection method on the basis of enhancements to YOLOv8, achieving a reduction of 1.6M in model parameters while attaining an accuracy of 86.3%. However, this method lacks sufficient accuracy in counting overlapping wheat ears and is prone to recognition errors for objects sharing similar shapes and colors with background. In this study, introduction of lightweight structures DySample and SCAM enabled improved the PSDS-YOLOv8 model to achieve an accuracy of 96.5% while reducing parameter count by 4.6M. Additionally, the model demonstrates exceptional performance in minimizing missed detections of irregular wheat ear targets at image edges (**Figure 10**) and accurately detects small, dense, and overlapping wheat ears despite challenges such as light variations, morphological differences, and overlapping occlusions (**Table 8**). Comparative experiments with different target detection models further validate superiority of the proposed model in terms of both accuracy and efficiency in wheat ear detection (**Table 6**).

Overall, despite the excellent performance of the proposed model in terms of detection accuracy and efficiency. However, the study still possesses some limitations. First, the high uniformity of data collection limits the model’s adaptability and robustness under different shooting angles. Moreover, the performance of the model needs to be optimized to cope with complex light changes and significant morphological differences of wheat ears. In addition, in the face of increasing growth stages, environmental conditions and plant variability in wheat fields, the existing methods may suffer from a lack of accuracy when dealing with different growth stages or individual differences within the same plot. Future studies may consider adopting richer data acquisition strategies, such as multi-angle and multi-height image acquisition, and combining video streaming techniques to enhance the generalization ability and accuracy of the model in complex environments, and to more effectively address the challenges of large-scale wheat field monitoring.

### 4.3 Different growth stages identification of wheat ear

This study also analyzed impact of various growth stages on recognition of wheat ears, revealing that wheat is most effectively recognized at the maturity stage (**Figure 12**), consistent with findings of Hasan et al. (2018). Meng et al. (2023) elucidated underlying cause of this phenomenon, attributing it to fuller leaves present during the filling stage, which may lead to wheat being misidentified as wheat ears. To further investigate this phenomenon, we conducted an in-depth analysis of the image characteristics of wheat ears at different growth stages. The morphology of wheat wheats at the maturity stage tends to be stabilized, the contrast between the wheats and the background is enhanced, and the features of the wheats are more obvious. This facilitates model’s ability to capture and accurately recognize ear information. In contrast, wheat ear features during the filling stage and earlier stages are more ambiguous and variable, lacking a stable morphology. During the full ripe stage, boundary between wheat ears and background becomes blurred, making clear distinction difficult and increasing detection challenge for model. Therefore, it is advisable to prioritize images of wheat at the maturity stage in training and application of the target detection model to enhance both accuracy and efficiency in recognition.

## 5 Conclusions and future work

In this study, a lightweight wheat counting method based on PSDS-YOLOv8 is proposed to address the problems of small size, dense distribution, and serious overlapping of wheat ears in UAV wheat images in complex field environments. Firstly, we optimize the structure of the model detection layer to improve the recognition ability of the model for small targets and introduce the SPD-Conv structure to effectively suppress the interference of confusing background, and finally integrate two lightweight modules, DySample and SCAM, to reduce the computational complexity. The experimental results show that the PSDS-YOLOv8 model improves the mAP50 and mAP50:95 of YOLOv8s by 2.8% and 4.4%, respectively, while the number of model parameters is reduced by 40.6%, which balances the detection accuracy and resource consumption of the model.

In order to improve the generalization ability of the model, three wheat datasets, UAV_wheat, GWHD, and WEDD, are applied for training and testing to enhance the model’s adaptability to different scenes. To verify the generalization performance of the model, the PSDS-YOLOv8 model was used to test wheat images with different fertility periods and different natural scenes. The results show that the model can accurately detect wheat spikes at different growth stages, especially the counting performance is optimized at the wheat maturity stage. The high performance can still be maintained when applying it to wheat spikelet data in different scenes, which proves that the PSDS-YOLOv8 model proposed in this paper has good generalization performance.

In future research work, we will focus on the lightweight design of the wheat counting method, aiming to facilitate the easy deployment of the model. In order to improve the counting performance, more appropriate density estimation methods will be used, especially when facing the task of counting wheat ears with complex background and dense targets. In addition, more diverse data types, such as dynamic data like video streams, will be introduced to realize the real-time wheat counting function. The ultimate goal is to improve the performance and utility of wheat counting technology to provide accurate and efficient support for agricultural production.

## Data Availability

The raw data supporting the conclusions of this article will be made available by the authors, without undue reservation.
